# Frauen in der Geschichte der Urologie – Pionierinnen zwischen Professionalisierung, (Un‑)Sichtbarkeit und Erinnerung

**DOI:** 10.1007/s00120-026-02904-6

**Published:** 2026-07-31

**Authors:** Julia Nebe, Friedrich Moll, Matthis Krischel

**Affiliations:** 1 Institut für Geschichte, Theorie und Ethik der Medizin, Centre for Health and Society, Heinrich-Heine-Universität Düsseldorf, Moorenstr. 5, 40225 Düsseldorf, Deutschland; 2 Deutsche Gesellschaft für Urologie, Düsseldorf, Berlin, Deutschland

Mit der Gründung der Arbeitsgemeinschaft Urologinnen im Rahmen des 73. Kongresses der Deutschen Gesellschaft für Urologie e. V. (DGU) im Jahr 2021 erhielt die Diskussion über die Sichtbarkeit von Frauen im Fach neue öffentliche Aufmerksamkeit [1]. Die Auseinandersetzung mit Geschlechterverhältnissen in der Urologie erscheint damit zunächst als Gegenwartsphänomen. Tatsächlich berührt sie jedoch grundlegende historische Fragen, die eng mit der Entstehung, Spezialisierung und Professionalisierung des Fachgebiets verbunden sind.

Ein vergleichbarer Perspektivwechsel wurde in den vergangenen Jahren bereits in anderen medizinischen Disziplinen vollzogen. Für die Zahnmedizin konnte gezeigt werden, dass trotz einer lange männlich dominierten universitären Fachkultur bereits früh wissenschaftlich tätige Zahnärztinnen existierten, deren Leistungen jedoch erst retrospektiv sichtbar wurden [2, 3]. Fachgeschichte erscheint damit nicht länger ausschließlich als Geschichte institutioneller Entwicklungen und medizinischer Innovationen, sondern zugleich als Geschichte von Teilhabe, Ausschluss und fachkultureller Erinnerung [4–8].

Auch die Geschichte der Urologie wurde bislang überwiegend als Geschichte männlicher Akteure erzählt. Diese Perspektive ist insofern einseitig, als die Urologie weder historisch noch gegenwärtig eine ausschließliche „Männerheilkunde“ darstellt [5–11]. Darauf verweist nicht zuletzt der seit jeher erhebliche Anteil weiblicher Patienten innerhalb des Fachs. Exemplarisch hierfür steht die von Gustav Simon (1824–1876) im Jahr 1869 durchgeführte weltweit erste Nephrektomie an der 46-jährigen Arbeiterin Margaretha Kleb – ein Eingriff, der zu den bekanntesten Bildquellen der Medizingeschichte zählt [12, 13].

## Kampf um Chancengleichheit

Die Frage nach Frauen in der Geschichte der Urologie wird im Kontext eines breiteren gesellschaftlichen Transformationsprozesses gestellt. Bereits in den 1870er-Jahren wurde der Ausschluss von Frauen aus akademischer Bildung zunehmend grundsätzlich infrage gestellt. So erklärte Hedwig Dohm programmatisch: „Die wissenschaftliche Bildung des Weibes ist kein Privilegium, sondern ein Menschenrecht“ [14]. Vor dem Hintergrund begrenzter Bildungs- und Berufsmöglichkeiten verbanden sich Forderungen nach höherer Bildung zunehmend mit dem Anspruch auf freie Berufswahl, gesellschaftliche Teilhabe und wirtschaftliche Eigenständigkeit [1–3, 6–8, 10, 11, 15–23].

In dieselbe Richtung zielten die Statuten des 1865 gegründeten Allgemeinen Deutschen Frauenvereins, der sich die Aufgabe setzte, „für die erhöhte Bildung des weiblichen Geschlechts und die Befreiung der weiblichen Arbeit von allen ihrer Entfaltung entgegenstehenden Hindernissen mit vereinten Kräften zu wirken“ [18]. Dennoch blieb Frauen der Zugang zum Hochschulstudium im Deutschen Kaiserreich lange verwehrt; erst zwischen 1900 und 1909 öffneten sich die deutschen Universitäten schrittweise für Studentinnen [3, 6, 8, 10, 15–17, 20–22, 24]. Auch danach bestanden strukturelle Hürden beim Zugang zu medizinischer Spezialisierung, fachärztlicher Professionalisierung und wissenschaftlicher Anerkennung [3, 6–8, 11, 19–21, 25].

Systematisch sichtbar wurden frühe Fachvertreterinnen der Urologie erstmals durch die 2009 an der Medizinischen Hochschule Hannover entstandene Dissertation von Jessica Peter zur Geschichte der ersten Urologinnen in Deutschland [11]. Unter Betreuung von Dirk Schultheiss entstand damit eine erste wissenschaftliche Annäherung an Medizinerinnen, die sich im Grenzbereich zwischen Urologie, Dermatologie und Venerologie bewegten. Einzelne dieser Biographien wurden im Rahmen späterer Forschungsprojekte zur Geschichte der deutschen Urologie im Nationalsozialismus sowie zu verfolgten jüdischen Ärztinnen erneut aufgegriffen. Exemplarisch zu nennen sind Dora Gerson (1884–1941), Johanna Hellmann (1890–1981) sowie Dora Brücke-Teleky (1879–1963), das erste weibliche Mitglied der DGU [6–8, 10, 11, 26, 27]. Mit der wissenschaftshistorischen Ausstellung der DGU auf dem Kongress 2022 in Hamburg erhielten diese Akteurinnen zusätzliche Sichtbarkeit [6–8, 10].

Trotz dieser Impulse bleibt die Forschungslage fragmentarisch. Insbesondere ist bislang nur gelegentlich systematisch reflektiert worden, nach welchen Kriterien Ärztinnen retrospektiv überhaupt der Urologie zugerechnet werden. Diese Frage besitzt für die frühe Fachgeschichte besondere Relevanz, da sich die Urologie erst vergleichsweise spät als eigenständiges Spezialfach institutionalisierte. Vor der Bremer Richtlinie von 1924 bestanden fließende Übergänge zwischen Urologie, Dermatovenerologie, Chirurgie, Innerer Medizin und Gynäkologie; Bezeichnungen wie „Facharzt für Harnleiden“ oder „Facharzt für Harnkrankheiten“ waren häufig und schlossen unterschiedliche fachliche Profile ein [25, 26, 28]. Zur Klassifikation früher Urologinnen und Urologen hat Julia Bellmann (2011) vier Kriterien vorgeschlagen, deren Aussagewert sukzessiv abnimmt: (1) Selbstidentifikation im Reichsmedizinaltkalender, ergänzend zu Bellmann sollten hier auch andere ausdrückliche Selbstidentifikationen z. B. auf Praxisschildern berücksichtigt werden, (2) Mitgliedschaft in einem Vorstand einer urologischen Vereinigung, Schriftleitung einer urologischen Publikation, Leitung einer urologischen Klinik, Forschungs- oder Lehrtätigkeit in der Urologie, (3) einfache Mitgliedschaft in einer urologischen Vereinigung, praktische Arbeit, die auch urologische Fälle einschließt, Teilnahme an urologischen Kongressen, Veröffentlichungen zu urologischen Themen, z. B. als Fallberichte und (4) Schwerpunktsetzung in medizinischen Praktika und Famulaturen, Wahl eines urologischen Dissertationsthemas [26].

## Den Blick systematisch auf Pionierinnen der Urologie lenken

Der vorliegende Beitrag eröffnet eine auf mehrere Veröffentlichungen angelegte Publikationsreihe zur Geschichte früher Urologinnen in Deutschland – welche wiederum an bereits erschienene einzelbiographische Beiträge anschließt [3, 6, 7, 10]. Im Zentrum der Publikationsreihe steht die Frage, wie Frauen an der Herausbildung und Professionalisierung der Urologie beteiligt waren? Unter welchen Bedingungen sie fachliche Anerkennung erlangen konnten? Und weshalb viele ihrer Leistungen in der fachhistorischen Erinnerung nur begrenzt sichtbar geblieben sind? Ziel ist es, ausgewählte Pionierinnen der Urologie auf der Grundlage historischer Quellen systematisch zu erschließen und ihre Biographien in den fach- und wissenschaftshistorischen Kontext einzuordnen. Auf diesen programmatischen Auftakt folgen Einzelstudien zu Elsbet Forßmann (1906–1993), Elisabeth Schmitz (1903–1973), Johanna Hellmann (1890–1981), Johanna Negendank (1880–?) und Meta Oelze-Rheinboldt (1893–?) ausführlich untersucht. Die einzelnen Biographien werden dabei nicht als isolierte Lebensgeschichten verstanden, sondern als vergleichbare Fallstudien, anhand derer sich unterschiedliche Formen weiblicher Teilhabe an Professionalisierungs‑, Spezialisierungs- und Erinnerungsprozessen innerhalb der Urologie analysieren lassen.

Methodisch verbindet die Untersuchung Elemente prosopografischer und kollektivbiografischer Forschung [29–35]. Prosopografische Verfahren ermöglichen die strukturierte Analyse gemeinsamer Merkmale historischer Personengruppen und schaffen die Grundlage für vergleichende Aussagen zu Bildungswegen, Karriereverläufen und institutionellen Einbindungen [29, 34, 35]. Zugleich werden Biographien nicht isoliert betrachtet, sondern als Ausdruck historisch variabler Möglichkeiten beruflicher Positionierung gelesen.

Der Beitrag knüpft damit an aktuelle Überlegungen zur Erinnerungskultur an. Denn fachhistorische Sichtbarkeit entsteht nicht allein durch individuelle Leistung, sondern auch durch Prozesse der Auswahl, Tradierung und nachträglichen Zuschreibung. In diesem Sinne verweist Aleida Assmann darauf, dass „nicht alles Vergangene erinnert und nicht alles Erinnerte Geschichte wird“ [30]. Der vorliegende Beitrag versteht sich daher als Beitrag zu einer um eine Geschlechterperspektive erweiterten Geschichte der Urologie und fragt danach, unter welchen Bedingungen Frauen fachliche Anerkennung gewinnen konnten – und weshalb einzelne Akteurinnen aus dem fachkulturellen Gedächtnis wieder verschwanden [6–8, 10].

Gerade für die frühen Urologinnen galten dabei häufig andere Voraussetzungen für berufliche Sichtbarkeit als für ihre männlichen Fachkollegen. Ihre Tätigkeit vollzog sich oftmals nicht primär im universitären oder wissenschaftlichen Raum, sondern in Klinik, Praxis und fachärztlicher Versorgung. Dennoch wirkten sie an der Etablierung und Ausdifferenzierung des Fachs mit und eröffneten neue berufliche Handlungsspielräume innerhalb eines lange männlich dominierten Spezialfachs [6–8, 10, 11].

Ein besonderes Augenmerk gilt dabei der Frage, welche Hürden diese Ärztinnen in ihrem beruflichen Umfeld zu überwinden hatten und welche Faktoren letztlich dazu beitrugen, dass sie innerhalb des Fachs Sichtbarkeit, Anerkennung und – in unterschiedlichem Ausmaß – dauerhafte Spuren hinterließen. Die folgenden Biographien werden daher nicht als Ausnahmegeschichten individueller Exzellenz gelesen, sondern als historische Fallbeispiele für unterschiedliche Wege weiblicher Teilhabe an Professionalisierungs‑, Spezialisierungs- und Erinnerungsprozessen innerhalb der Urologie.

## Fünf Protagonistinnen

Zu diesen frühen Vertreterinnen gehört die 1906 geborene Elsbet Forßmann (geb. Engel), die in der fachhistorischen Erinnerung häufig auf ihre Rolle als Ehefrau des Nobelpreisträgers Werner Forßmann (1904–1979) reduziert wurde, deren berufliche Tätigkeit jedoch darüber hinausweist. Nach ihrer Ausbildung bei der universitär ausgewiesenen Fachgröße für Urologie Prof. Karl Heusch (1894–1986; [28, 36, 37]) am Rudolf-Virchow-Krankenhaus in Berlin (1933–1934) gehörte sie in der frühen Bundesrepublik zu den ersten Ärztinnen, die den Facharzt für Urologie erwarben. Auf Grundlage einer breiten chirurgischen Qualifikation deckte sie das gesamte Spektrum des Faches ab und blieb der Urologie lebenslang verbunden. Elsbet Forßmann zählt damit zu den frühen Pionierinnen der bundesrepublikanischen Urologiegeschichte [11].

Ebenfalls zu diesen Ausnahmen gehörte die 1903 geborene Elisabeth Schmitz (geb. Schmitz), die sich früh konsequent urologisch spezialisierte. Ab 1931 absolvierte sie ihre Fachausbildung an der chirurgisch-urologischen Abteilung des Kaiserin-Augusta-Viktoria-Krankenhauses in Berlin-Lichtenberg beim im Nationalsozialismus als „jüdischstämmig“ verfolgten Urologen Joachim Stutzin (1878–1954; [7, 26, 27]). Ihre 1932 publizierte Arbeit zur „vaginalen Ureterpalpation“ belegt die frühe wissenschaftliche Auseinandersetzung mit urologischen Fragestellungen. 1934 erhielt sie wohl als eine der ersten Frauen in Deutschland die ausschließliche Facharztanerkennung für Urologie und ließ sich im selben Jahr als eine der ersten hauptberuflich urologisch tätigen Ärztinnen in Wuppertal-Barmen nieder [11].

Johanna Hellmann, geboren 1890 in Nürnberg, zählt ebenfalls zu den frühen Vertreterinnen des Faches. 1915 approbiert, erhielt sie ihre prägende chirurgische Ausbildung an der Chirurgischen Universitätsklinik Kiel bei Willy Anschütz (1870–1954; [11, 28, 36–38]), einem wichtigen Akteur der frühen Entwicklung der Urologie im deutschsprachigen Raum, und erwarb dort eine breite operative Qualifikation einschließlich urologischer Tätigkeitsfelder. In zeitgenössischen Selbstzuschreibungen, etwa im Reichsmedizinalkalender [39], führte sie neben der Chirurgie ausdrücklich auch die Urologie, während sie in der Medizingeschichte überwiegend als Chirurgin verankert blieb [11, 37, 40].

Demselben erweiterten fachlichen Umfeld gehörte die 1880 geborene Johanna Negendank an. Nach einem später nachgeholten Reifezeugnis studierte sie Medizin in Freiburg, Bonn, Leipzig und München. Ihre erste ärztliche Tätigkeit führte sie 1915/16 an die dermatologische Abteilung des Krankenhauses Dresden-Friedrichstadt, das aufgrund seiner urologischen Tradition als frühes Schnittstellenmilieu dermatologisch-urologischer Versorgung gilt. Ab den 1920er-Jahren ist sie in Chemnitz als Dermatologin niedergelassen und zugleich als Fachärztin für Haut- und Geschlechtskrankheiten sowie Urologie ausgewiesen, was auf eine urologische Zusatzqualifikation im Rahmen ihrer Praxis hinweist. Johanna Negendank steht dabei exemplarisch für all jene Medizinerinnen, die sich vor der Bremer Bremer Richtlinien von 1924 im Grenzgebiet zwischen Urologie, Dermatologie und Venerologie bewegen [8, 11].

Einen stärker wissenschaftlich geprägten Zugang zu diesem erweiterten fachlichen Feld verkörpert Meta Oelze-Rheinboldt (née Rheinboldt), geboren 1893. Nach dem Medizinstudium und der Promotion in Heidelberg durchlief sie klinische Stationen in Chirurgie, Innerer Medizin, Gynäkologie, Orthopädie und insbesondere Dermatologie. Seit den 1920er-Jahren trat sie mit einer Vielzahl wissenschaftlicher Arbeiten zu dermatologisch-urologischen und gynäkologischen Fragestellungen hervor. Im Reichsmedizinalkalender führte sie die Facharztbezeichnungen Haut- und Geschlechtskrankheiten sowie Urologie [41]. Im Unterschied zu Negendank erscheint Urologie bei Oelze-Rheinboldt weniger als praxisbezogene Erweiterung einer dermatologischen Tätigkeit denn als eigenständiger wissenschaftlicher Schwerpunkt innerhalb eines weiterhin interdisziplinär geprägten Arbeitsfeldes [11].

## Schlussfolgerung

Die Geschichte der Urologie lässt sich nur dann angemessen verstehen, wenn neben den etablierten Protagonisten auch jene Akteurinnen in den Blick genommen werden, die an der Entwicklung des Fachs beteiligt waren, deren Beiträge jedoch lange Zeit nur begrenzt wahrgenommen wurden. Die geplante Publikationsreihe möchte dazu beitragen, diese Forschungslücke zu schließen und frühe Urologinnen als Teil der Professionalisierungs‑, Spezialisierungs- und Erinnerungsgeschichte der Urologie sichtbar zu machen. Die folgenden biographischen Studien verstehen sich dabei nicht als nachträgliche Würdigung einzelner Ausnahmefiguren, sondern als Bausteine einer differenzierten und um eine Geschlechterperspektive erweiterten Fachgeschichte und fachkulturellen Erinnerung.

## Data Availability

Alle dieser Arbeit zugrunde liegenden Daten sind in diesem Artikel enthalten.
